# Nanogroove-Induced Enhancement of Neural Spike Activity in Stem Cell-Derived Networks

**DOI:** 10.3390/mi17050524

**Published:** 2026-04-25

**Authors:** Rahman Sabahi-Kaviani, Marina A. Shiryaeva, Regina Luttge

**Affiliations:** 1 Neuro-Nanoscale Engineering, Department of Mechanical Engineering/Microsystems and Institute of Complex Molecular Systems, Eindhoven University of Technology, 5600 MB Eindhoven, The Netherlands; r.sabahi.kaviani@tue.nl (R.S.-K.); m.a.shiryaeva@vu.nl (M.A.S.); 2 Eindhoven Artificial Intelligence Systems Institute and Casimir Institute, Eindhoven University of Technology, 5600 MB Eindhoven, The Netherlands

**Keywords:** Nervous System-on-Chips (NoCs), nanogrooves, microelectrode array (MEA), neuroelectrophysiology, neural network organization, Ngn2 iNeurons

## Abstract

Nanogrooves provide instructive cues to cells in culture. Several nanofabrication techniques have been developed to create biomimetic substrates, advancing our understanding of cell adhesion. Their integration into nervous system models highlights the critical role of the extracellular matrix (ECM) in developing functional tissue constructs for in vitro platforms such as Brain-on-Chip (BoC) and Nervous System-on-Chip (NoC). This study presents a nanofabrication approach that integrates photolithography and microtransfer molding (μTM) to pattern nanogrooves using photocurable polymer NOA81 onto microelectrode array (MEA) plates. The resulting nanogrooves exhibited a pattern periodicity of 976 nm and a ridge width of 232 nm, as confirmed by scanning electron microscopy and atomic force microscopy. We assessed the biocompatibility and functional impact of these modified substrates using human induced pluripotent stem cell (hiPSC)-derived neuronal cultures. Neurons cultured on nanogroove-modified MEAs exhibited aligned neural processes due to the anisotropic surface features and expressed vivid spiking behavior and higher burst frequency compared to randomly cultured neuronal networks. In conclusion, the proposed fabrication technique integrates nanogrooves with commercial MEAs using a combination of microtransfer molding and photolithography, resulting in modified culture substrates that enhance spike activity and network organization, aiding in the development of more in vivo-like neural models.

## 1. Introduction

Micro- and nanofabrication techniques have been shown to facilitate the development of innovative in vitro brain models, i.e., Brain-on-Chips (BoCs), mimicking aspects of the brain’s complex microenvironment [1,2,3]. These models have recently been utilized to improve our understanding of brain functions and associated diseases, but also to enable novel treatments and medications extending such models for diseases of the human nervous system [4]. Additionally, the integration of biomimetic microenvironments in models of the nervous system has demonstrated relevance [5]. Nervous System-on-Chip (NoC) technology aims to develop a platform for studying neural functions, drug testing, and disease modeling across different regions of the nervous system by incorporating multiple cell types into three-dimensional (3D) constructed neural networks [6,7,8,9]. Recent work has further demonstrated that microfabricated platforms combining 3D neuronal constructs with microchannel-guided neurites and optical interfaces can mimic pathway-like neuronal activation, enabling patterned stimulation and investigation of cortical information processing in vitro [10].

The extracellular matrix (ECM) is a key component of such engineered microenvironments, as it plays a crucial role in cell differentiation and maturation [11,12,13]. Inspired by the molecular structure of cell–cell interfaces in nature [14], designed surface nanotopographies have emerged as a tool to manipulate cellular behavior and function in a culture dish simply by modulating the geometrical features that influence the cells’ development [9,15,16]. Nanotopographic patterns can promote neurite outgrowth and accelerate neuronal differentiation. For example, neurons cultured on nanopillars exhibit fewer neurites, faster elongation, and earlier axon specification than those grown on flat surfaces, demonstrating how the physical environment can affect neuronal maturation [17].

Nanogrooves, in particular, have been shown to enforce neurite spreading during differentiation to align with the grooves’ direction in various neuronal cell culture models such as the neuroblastoma cell line SH-SY5Y, primary cells, and hPSC-derived neurons [18,19,20,21,22,23,24]. In some of these cultures, recordings of neuronal activity revealed an increased spike rate [23,25]. Leveraging surface nanotopography in innovative assay technologies has the potential to enable breakthroughs in stem cell–based in vitro neuronal modeling. To this end, we present a new fabrication method for integrating nanogrooves with microelectrode array (MEA) technology and validating their influence on neuronal activity.

A review of the state of the art in substrate modification with nanotopography for cell culture reveals the use of various materials, including polystyrene [26,27], glass and imprinted resist [18], silicon [28], polydimethylsiloxane [18,29], as well as cyclic olefin copolymer [30]. Each of these patterned substrates exhibits distinct mechanical and chemical properties that consequently influence cellular responses. Furthermore, it has even been demonstrated that the alignment of neurite outgrowths can extend into the third dimension beyond the nanopatterns at the surface, when cultures were carried out in ECM mimicking microenvironments like Matrigel atop of the cells seeded onto the nanogrooves [31].

We have recently demonstrated increased neuronal activity on nanogrooved substrates using calcium imaging as the primary readout [23]. However, to systematically study functional maturation through electrical signals, it is desirable to fabricate these nanotopographic structures directly on MEA plates, as the measurement of electrical activity is a fundamental tool for characterizing neural networks [32].

Understanding the functional behavior of differently designed neural cell networks remains challenging, prompting continuous improvements in MEA platforms to address this need. To contribute to this development, we demonstrated that microtransfer molding [33] can be combined with photolithography to modify the surface of MEA plates with nanogrooves. Specifically, we developed a fabrication process using UV-curable Norland Optical Adhesive 81 (NOA81, Norland Products Inc., Jamesburg, NJ, USA) to pattern nanogrooves directly onto MEA plates. We then investigated the ability of these nanogroove-modified MEA plates to record neural activity from a human induced pluripotent stem cell (hiPSC)-derived neuronal culture. In this study, we built on an Ngn2+ induced neuronal culture protocol [34] to introduce anisotropic organization into the neural networks and observed increased spike rates in this NoC model.

## 2. Materials and Methods

### 2.1. hiPSC-Derived Cultures

Human WTC-11 stem cells, stably overexpressing Ngn2 (a kind gift of Dr. Nadif Kasri), were expanded and differentiated towards cortical excitatory neurons as previously described [34]. In brief, the cells were thawed and precultured on Matrigel (#356231, Corning Incorporated, Corning, NY, USA) for one passage before starting the neuronal induction in our experiments. Prior to cell seeding, all MEAs were sterilized by exposure to UV light for 10 min, followed by treatment with isopropanol (IPA, Article number 76051455.9010, Boom B.V., Meppel, The Netherlands) for 10 min [35]. The utilization of IPA instead of ethanol for cleaning and sterilization processes is recommended in NOA81-based cultures due to the observed swelling of NOA81 in the presence of ethanol [36]. The cell-toxic isopropanol was removed by 5 times washing with Milli-Q water. MEAs were coated sequentially from solution using poly-L-ornithine (PLO, P3655, Merk KGaA, Darmstadt, Germany) dissolved in 50 mM borate buffer (28341, Thermo Fisher Scientific, Waltham, MA, USA) to a concentration of 50 μg/mL and laminin (L2020, Merk KGaA, Darmstadt, Germany) diluted in cold DMEM/F12 (11320033, ThermoFisher Scientific, Waltham, MA, USA) to a final concentration of 20 μg/mL. MEAs were incubated with the PLO for 3 h at 37 °C and then washed 3 times with Milli-Q water. Immediately after full aspiration of the liquid in the last washing step, laminin solution was added. The MEAs covered with laminin solution were sealed with parafilm and incubated overnight at 4 °C. The Ngn2+ hiPSCs were plated on MEAs with a 10,500 cells/cm^2^ cell density on 0 day in vitro (0 DIV), and the neuronal induction was initiated and continued until 21 DIV. To improve the neuronal functionality and surface attachment, the culture conditions described in Frega et al. [34] were adapted by replacing 50% of the Neurobasal medium with astrocyte conditioned medium (ACM, 1811, ScienCell Research Laboratories, Carlsbad, CA, USA) [35]. All neuronal cultures used in this study were derived from the same passage number to ensure consistency across experimental conditions. The fabrication details of topographically modifying the MEA plates are described in Section 2.3. In total, four MEA plates were used in this study, each representing a distinct experimental condition. Each MEA was seeded with one independent hiPSC-derived neuronal culture, resulting in one biological replicate per condition. The aim of this work was to validate the fabrication workflow and evaluate the feasibility of recording neural activity on nanogrooved MEAs; therefore, the study was not designed for statistical comparison across multiple biological replicates.

### 2.2. Immunostaining and Imaging

The fixation and staining procedures in Ngn2+ iNeurons were performed as described in the literature [37,38]. In short, the neurons were fixed by 4% PFA with 4% sucrose for 15 min, and permeabilized by treatment with 0.2% Triton X-100 for 10 min. Further, the cells were incubated in a blocking solution (5% normal goat serum in DPBS) for 1 h and incubated with primary antibodies at 4 °C overnight. The applied primary antibodies: mouse anti-Neurofilament Marker pan axonal cocktail (antibody SMI-312, 1:500 dilution factor, 837904, BioLegend, Inc., San Diego, CA, USA) and Rabbit anti-MAP2 antibody (1:100 dilution factor, ab32454, Abcam, Cambridge, UK). The next day, the cells were incubated with secondary antibodies for 1 h, followed by counterstaining with 1 µg/mL DAPI for 3 min; the applied mixture of secondary antibodies: goat-anti-mouse Alexa 488 (A-11029, Invitrogen, Carlsbad, CA, USA), and goat-anti-rabbit Alexa 647 (A-21245, Invitrogen, Carlsbad, CA, USA). One MEA per topographical condition was stained for immunofluorescence analysis, resulting in a total of four stained samples. The cells were imaged using a fluorescence microscope (Leica DMi8 fluorescence microscope, Wetzlar, Germany) with Leica Application Suite X (LAS X) imaging and analysis software. The images showing neuronal distribution across MEAs were captured using the tiling capability of the Leica DMi8, which assembles multiple images into a mosaic of tiles. A 20× objective lens was used for capturing these images, achieving a resolution of approximately 0.33 µm per pixel. Additionally, images showing localized neuronal growth on the MEAs were captured using a 10× objective lens, with a resolution of approximately 0.66 µm per pixel.

### 2.3. Topographically Modifying MEA Plates

**Figure 1:**
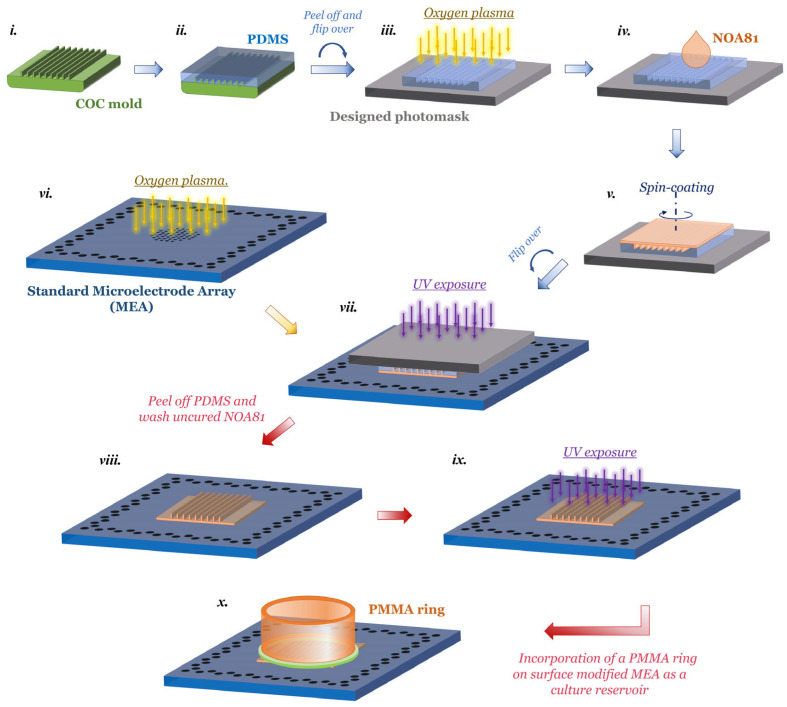
Schematic representation of the nanofabrication process flow, adapted and extended from [33]. (**i**) COC nanogroove master mold as previously provided by Bastiaens et al. [23]; (**ii**) replication of nanogrooves from the master mold to a PDMS mold; (**iii**) placement of the PDMS mold on the back side of the photomask, followed by oxygen plasma treatment to render the surface hydrophilic; (**iv**) dispensing NOA81 liquid on the PDMS working mold; (**v**) spin-coating NOA81 on the mold; (**vi**) activation of MEA surface via oxygen plasma treatment; (**vii**) transfer of the NOA81 film onto the MEA substrate by bringing the coated PDMS mold into contact, followed by alignment of MEA electrodes with the photomask features and UV exposure for curing; (**viii**) peeling of the PDMS mold and removal of uncured NOA81; (**ix**) additional UV exposure to fully cure the NOA81 layer on the MEA substrate; and (**x**) integration of a PMMA ring onto the modified MEA to serve as a culture reservoir.

We prepared nanogroove-modified MEA plates as illustrated in Figure 1, outlining the essential steps of the nanofabrication process, including the fabrication of a polydimethylsiloxane (PDMS) soft template from a cyclic olefin copolymer (COC) mold according to Bastiaens et al. [23,29]. In brief, the COC master mold was prepared using a quartz master in jet and flash imprint lithography (J-FIL) to pattern nanoresist onto a standard silicon wafer. The nanoresist patterns were then transferred to the COC using thermal nanoimprint lithography, creating a negative copy. This process resulted in a COC mold with the desired nanogroove features. Following this, PDMS was spin-coated on the COC master mold at 500 rpm for 30 s with an acceleration of 200 rpm/s and cured in a 65 °C oven for 4 h prior to peeling it from the COC template, resulting in PDMS nanogrooves with a thickness of approximately 130 μm (Figure 1i,ii).

To incorporate nanogrooves on MEAs, we used Norland Optical Adhesive 81 (NOA81, Norland Products Inc., Jamesburg, NJ, USA), which has been previously used for creating microdevices [39,40,41,42,43]. The process combined microtransfer molding (μTM), previously reported by us [33], and photolithography. In this work, a 15 mm × 15 mm PDMS template was flipped over, mounted, and aligned with the 120 electrode designs on a foil photomask (Selba S.A., Versoix, Switzerland). Details of the foil photomask design can be found in Supplementary Materials S1.

Since Bastiaens et al. [23] showed that nanogrooves with a pattern periodicity of 1000 nm and a ridge width of 230 nm influenced SH-SY5Y cell neurite alignment the most, we selected a PDMS template mold to create similar patterns. This nanopattern mold has a pattern periodicity of 1000 nm and a ridge width of 780 nm. Two specific orientations of the nanogrooves relative to the arrangement of the electrodes were selected: 90° alignment (NG-90 MEA, with nanogrooves running parallel to the electrodes) and 45° alignment (NG-45 MEA, with nanogrooves running diagonally across the rows and columns of electrodes). Figure S2 in the Supplementary Materials shows schematic drawings of these configurations. Additionally, a flat PDMS mold was used to create a flat NOA81-MEA as a control experiment.

The PDMS mold was then made hydrophilic by oxygen plasma treatment using an EMITECH K1050X plasma asher (Quorum, Laughton, UK) at 3 W for 30 s (Figure 1iii). Next, an excess of NOA81 liquid was poured onto the PDMS mold and spin-coated at (a) 500 rpm for 10 s at an acceleration rate of 200 rpm/s, followed by (b) 8000 rpm for 30 s at an acceleration of 1200 rpm/s, and finally, (c) a deceleration rate of 1200 rpm/s resulting in a thickness typically in the range of 2–4 µm (Figure 1iv). The surface of the MEA without a culture reservoir, i.e., 120MEA200/30iR-Ti-w/o plate (Multi Channel Systems MCS GmbH, Reutlingen, Germany), was also activated in the same plasma asher with the settings of 100 W and 90 s, prior to the transfer step (Figure 1v).

Next, the activated MEA and the photomask-PDMS-NOA81 assembly were aligned with the corresponding features in a mask aligner (MJB4 Mask Aligner, SÜSS MicroTec SE, Garching, Germany) and subjected to ultraviolet (UV) exposure energy of 670 mJ/cm^2^ (Figure 1vi). Subsequently, the photomask-PDMS mold assembly was carefully peeled from the MEA, and the MEA was then washed with acetone and isopropanol and dried with an air gun (Figure 1vii). If additional washing/drying steps were required, this process was repeated. Consequently, the MEA was subjected to additional UV light exposure via the UV-LED exposure system (IDONUS, UV-EXP 150R, Neuchatel, Switzerland) with an energy dosage of 8000 mJ/cm^2^ at an intensity of 15 mW/cm^2^ to fully cure the NOA81 film (Figure 1viii). Details of the mask aligning and washing/drying steps are in the Supplementary Materials S3.

This process yields a nanotopographical pattern on top of a plasma-activated MEA plate and at the same time allows cells direct access to the electrodes by means of photolithographic microscale patterning of the microtransfer molded NOA81.

Finally, based on the dimensions of the glass ring on the bare 120MEA200/30iR-Ti-gr, we made a PMMA ring and affixed it to our surface-modified MEAs (Figure 1ix), details of which are described in Supplementary Materials S4. The MEA assembled with a PMMA ring is displayed in Figure S6a in the Supplementary Materials.

### 2.4. Scanning Electron and Atomic Force Microscopy of Nanogrooves

The transferred nanogrooves were examined using a QUANTA 600 F scanning electron microscopy (SEM) tool (FEI, Eindhoven, The Netherlands) to capture images of the nanogrooves in low vacuum mode, with a 5 kV acceleration voltage, spot size of 4.0, and a working distance of 9.8 mm. Moreover, to study the surface topography in greater detail, an atomic force microscope (AFM) was used. The topographical data were obtained and recorded using an XE-100 AFM tool (Park Systems Corporate, Suwon, Republic of Korea) in tapping mode, with a noncontact cantilever (PPP-NCHR, Park Systems Corporate, Suwon, Republic of Korea). The scan resolution was set to 256 × 256 pixels, and the scan area was 6 µm × 6 µm, with a scan rate of 1 Hz for high-resolution scanning. The XEP software (Park Systems Corporate, Suwon, Republic of Korea) was used to operate the AFM tool, while GWYDDION software (Version 2.60) [44] was used to analyze the captured data.

### 2.5. Alignment Analysis

To evaluate the orientation of the nanogrooves incorporated on MEAs and how the nanogrooves affected the guidance of Ngn2+ iNeurons, the captured images of fabricated devices and the cell cultures were analyzed using the FIJI image analysis software (Version 2.16.0) [45]. The latter was performed with the Directionality Analysis plugin. This analysis generates histograms of feature orientations in the images to determine if there was any preferred orientation of cellular features. The microscopy field of view for each image that was analyzed was approximately 1.4 mm × 1.4 mm, covering both the electrode area and the nanogroove region on the MEA. The MEA electrode tracks were not excluded from the analysis; however, their contribution to the orientation histogram was minimal, as the transform primarily responds to the spatial frequency content of finer cellular features. To independently validate the observed neurite alignment and to address potential bias from the oriented MEA electrode traces inherent to Fourier-based analysis, a complementary structure-tensor-based orientation analysis was performed using the OrientationJ plugin in FIJI. For each MEA with a specific topography, only one image was analyzed, and there was only one sample for each MEA with a specific topography.

### 2.6. Electrical Readout and Signal Analysis

To enable the study of how nanogrooves influence the activity of the cultured neuronal networks in different experimental conditions, MEA recordings at day 18 were taken using the MEA-2100 System (Multi Channel Systems MCS GmbH, Reutlingen, Germany). This system consists of a hardware amplifier and acquisition unit for up to 120 electrodes simultaneously. The temperature of the MEA plate was regulated to 37 °C, and five successive measurements were taken from each of the four plates with a duration of 1 min for each measurement, with a 5 min delay prior to the first measurement. Thus, spiking analysis was performed on all four MEA samples, one representing each topographical condition. The recorded electrophysiological signals were then assessed and analyzed by a Multi Channel Analyzer (Multi Channel Systems MCS GmbH, Reutlingen, Germany). The software configuration is outlined in Supplementary Materials S5. Following acquisition, data post-processing was performed by a Notch filter at 50 Hz in series with a second-order Butterworth high-pass filter at a cut-off frequency of 200 Hz. Examples of the filtered data can be found in Supplementary Materials S6. The predominant feature of neuronal cell culture firing behavior is the occurrence of spikes. These are spontaneous discharges of action potentials that transpire without the influence of any external stimulation [46]. To detect spikes, a voltage threshold of five times V_noise __−__ rms_ was utilized [47,48]. To determine the bursts, which occur when these spikes become more synchronized, certain features of the spikes, such as the interspike interval, need to be considered [49]. Synchronized bursts that occur throughout the network are referred to as network bursts [49], and a minimum threshold of spontaneous channels is necessary for their recognition. In this study, the minimum threshold was defined as half the total number of bursting channels, and the parameters for burst [50] and network burst recognition (Multi Channel Analyzer software manual, V 2.20.15) were as follows:

• Maximum interval to start burst: 50 ms;
• Maximum interval to end burst: 50 ms;
• Minimum interval between bursts: 100 ms;
• Minimum duration of bursts: 50 ms;
• Minimum number of spikes in bursts: 4;
• Minimum active channels: 10;
• Minimum spontaneous channels: 5.

### 2.7. Statistical Analysis of Electrophysiological Recordings

The electrical signals generated by cell cultures on each MEA were recorded over a period of one minute, with five consecutive measurements taken. The resulting data was expressed as an average (avg) value ± standard deviation (SD). A one-way analysis of variance (ANOVA, two-sided) was employed to determine whether there was a statistically significant difference between the means of the different MEA culture conditions. For the *p*-values < 0.05, post hoc tests were conducted with a significance level of *p* = 0.05 and *p* = 0.01 to determine which groups exhibited significant differences. Statistical analysis was performed using the “statsmodels” and “scipy” modules in an in-house programmed Python code.

## 3. Results and Discussion

### 3.1. Combined Micro- and Nanofabrication Process for Nanogroove-Modified MEA Plates

**Figure 2:**
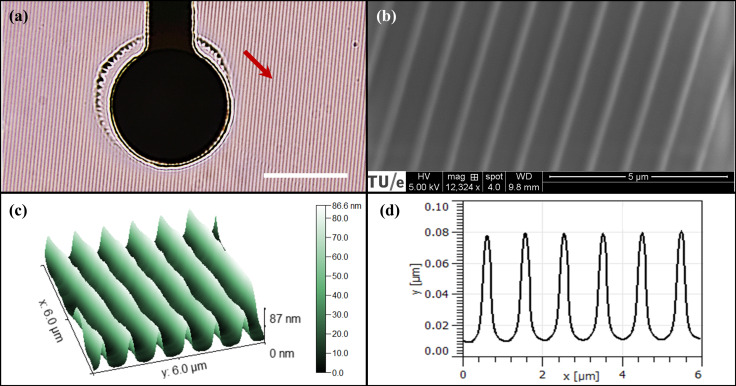
Structural characterization of NOA81 nanogrooves. (**a**) Optical image of NOA81 nanogrooves incorporated around an electrode of an MEA. The grooves are clearly visible, as indicated by the arrow (scale bar 20 μm). (**b**) SEM micrograph, (**c**) AFM topographical scan, and (**d**) the respective cross-sectional line profile of the NOA81 nanogrooves transferred from the PDMS mold with a pattern periodicity of 1000 nm and a ridge width of 780 nm.

We examined the functional behavior of hiPSC-derived neural networks on NOA81 nanogrooves. To this end, we successfully applied a combination of photolithography and μTM to modify commercial MEA plates with nanogrooves. The presence of nanogrooves on the MEA surface was confirmed through visual inspection under a microscope (Figure 2a). The microscopic images of a single electrode on an MEA incorporated with NOA81 nanogrooves confirmed that only the area between the electrodes was covered by the nanopatterned NOA81. Two specific configurations relative to the arrangement of the electrodes resulted from the alignment process, namely 90° alignment between nanogrooves and the rows of the electrode array (sample name refers to NG-90) and 45° alignment between nanogrooves and columns of the electrode array, with nanogrooves running diagonally across the electrodes (sample name refers to NG-45). Supplementary Materials S7 provides further processing details and optical micrographs of the resulting devices and structures.

In our previous works, we systematically characterized each step of the fabrication chain, COC master mold, PDMS working mold, and NOA81 nanogroove films, using SEM, AFM, and optical interference/diffraction analysis, and demonstrated high pattern transfer fidelity and repeatability across multiple transfers [33]. In the present study, we build on this validated process and focus on demonstrating that the same nanogroove geometry can be reliably integrated onto MEA plates and used for functional electrophysiological readout.

### 3.2. Nanogroove Characterization on the Nanogroove-Modified MEA Plates

Figure 2b shows a SEM micrograph, and Figure 2c shows an AFM scan with a measurement resolution set to 256 × 256 pixels over an area of 6 × 6 µm^2^. Figure 2d depicts a cross-sectional line profile of the received pattern. A pattern periodicity of 976 ± 16 nm can be inferred from Figure 2d, with a ridge width of 232 ± 6 nm and a ridge height of 68.2 ± 0.9 nm (*n* = 4). In this context, “*n*” denotes four consecutive grooves measured within the same AFM line profile. Pattern periodicity, ridge width, and ridge height refer to the distance between ridges, the full width at half height, and the peak-to-peak amplitude of the line profile, respectively. These measurements were performed on one representative nanogroove-modified MEA sample. The results indicate that the geometry of the NOA81 nanogroove closely approximates that of the PDMS mold. The specific pattern was selected because it previously elicited an alignment effect in SH-SY5Y and hiPSCN cultures reported by Bastiaens et al. [23].

### 3.3. Immunostaining Analysis of Ngn2+ iNeurons on NOA81-Modified MEA

**Figure 3:**
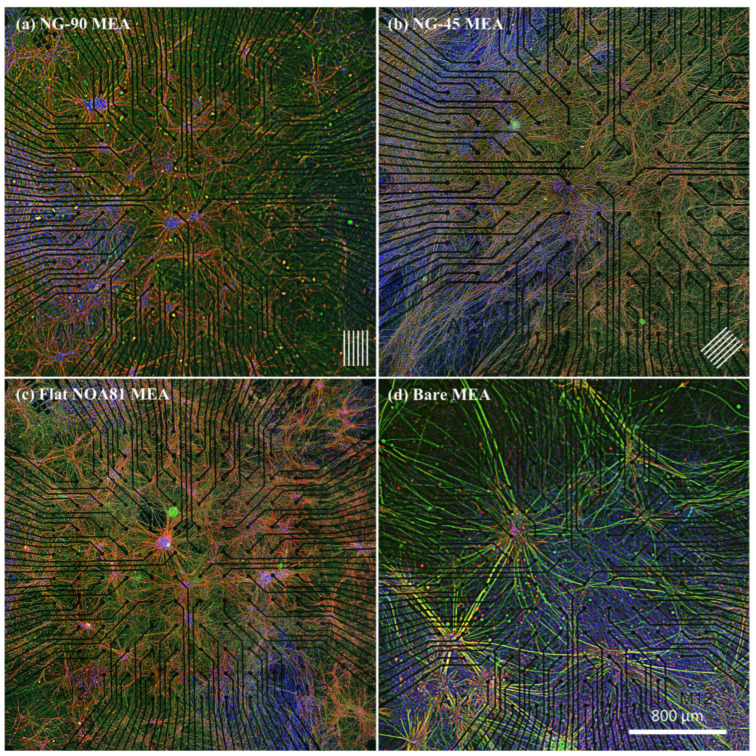
Neuronal distribution across MEAs with different topographies. Immunostaining images of cultured Ngn2+ iNeurons at 21 DIV covering the whole electrode array. Optical micrographs depict cells on (**a**) an NG-90 MEA and (**b**) an NG-45 MEA configuration, respectively (The arrays of lines at the bottom right indicate the direction of the grooves). Further optical micrographs depict cells on (**c**) an MEA incorporated with flat NOA81, and (**d**) a bare MEA, respectively. Red staining indicates microtubule-associated protein 2 (MAP2) of neurons, green staining indicates axonal neurofilament marker (SMI 312), and blue staining indicates the cell nuclei (DAPI).

Cultured Ngn2+ iNeurons resulted in a well-established reductionist (no glia cells) stem cell-derived neural network on each of the four seeded MEA plates. In contrast to the relatively short and low number of neurites determined in SH-SY5Y cell cultures [23,29,31,51], the neuronal processes of these cells extended several hundred micrometers in several days, forming large bundles of outgrowths between clusters of cells (Figure 3). Regardless of the configuration (bare or NOA81-modified MEA plates), cells differentiated into mature neurons after 21 DIV as depicted by the immunostaining images being positive for axonal neurofilament marker (SMI 312, green) and neuronal microtubule-associated protein marker (MAP2, red) (Figure 3a–d). To provide a reference for neuronal morphology under standard conditions, control cultures of Ngn2+ iNeurons grown on conventional substrates are presented in Supplemental Materials S8, showing comparable neuronal maturation and marker expression. It was noticed that cells migrated and formed small clusters, unlike in the SH-SY5Y cell culture, where 2D cultured cells are more spread out and can be observed as individual cells in the network of cells more easily [23,29,31,51].

### 3.4. Neuronal Network Organization

**Figure 4:**
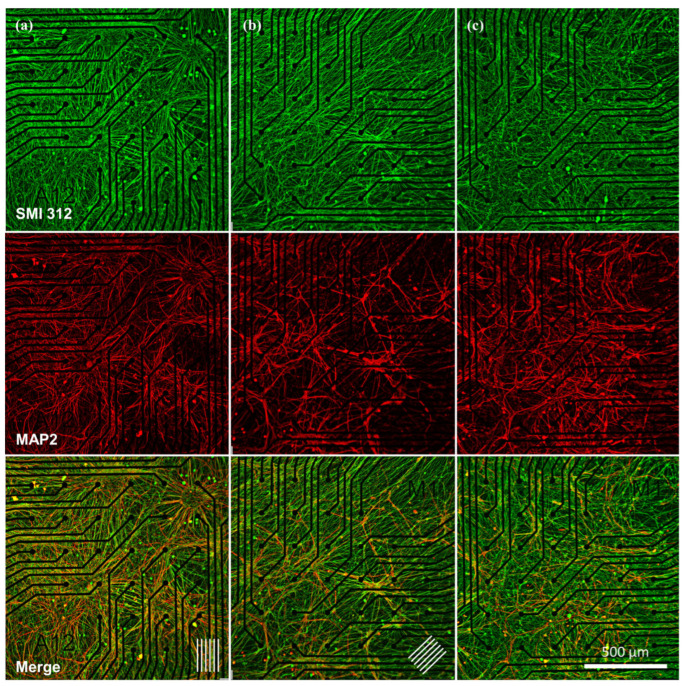
Localized neuronal growth on MEAs with varying topographies. Immunostaining images of cultured Ngn2+ iNeurons at 21 DIV covering a quarter of the surface where electrodes are (**a**) in MEA incorporated with NOA81 nanogroove in 90° orientation, (**b**) in 45° orientation (where the arrays of lines at the bottom right indicate the direction of the grooves), and (**c**) in the MEA incorporated with flat NOA81. Green staining indicates axonal neurofilament marker (SMI 312) and red staining indicates dendritic microtubule-associated protein 2 (MAP2) in neurons (scale bar 500 μm).

**Figure 5:**
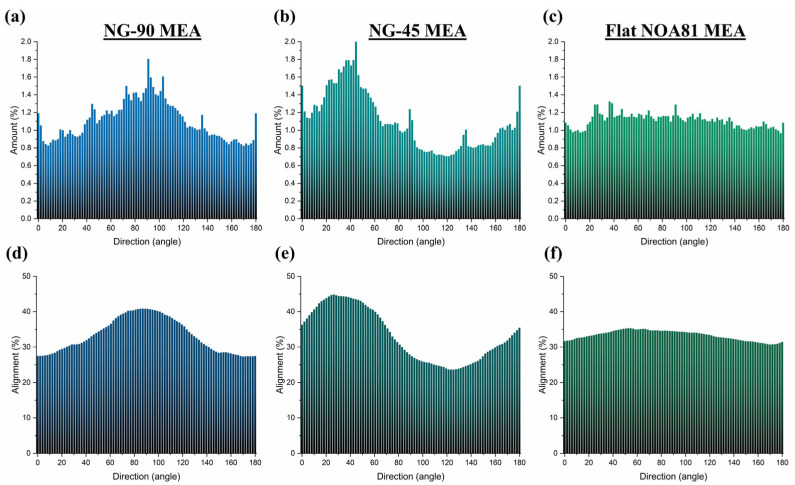
Directional neurite growth analysis on MEAs. The distribution of the neuronal extension in different directions for the whole area of electrode regions for the Ngn2+ iNeuron cultures on (**a**) NG-90 MEA, (**b**) NG-45 MEA, and (**c**) flat NOA81 MEA (with the average amount of 1.1%). Furthermore, the alignment distribution of the neuronal extension in different directions for the whole area of electrode regions for the Ngn2+ iNeuron cultures on (**d**) NG-90 MEA, (**e**) NG-45 MEA, and (**f**) flat NOA81 MEA. The percentage of neurites aligned at a specific angle is considered for the total amount of neurite within the range of ±30° with respect to that specific angle (with the average alignment of 33.3%).

Immunostained images (Figure 4a–c) stained by SMI 312 reveal that the alignment of axons is preferentially in the direction of the nanogrooves. However, no such observation was derived from MAP2 staining of the dendrites. These qualitative observations of axonal organization are further confirmed quantitatively by the histograms depicted in Figure 5a–c, respectively (*n* = 1). The preferential alignment of axons observed here is consistent with the structural organization of neural tissues, where oriented growth supports directional connectivity and efficient signal propagation. Reproducing such alignment in vitro is therefore important for enhancing the physiological relevance of engineered neural networks. To validate that the observed preferential alignment is not influenced by the oriented MEA electrode traces inherent to Fourier-based analysis, an independent structure-tensor-based orientation analysis (using the OrientationJ plugin, FIJI) was additionally performed and yielded qualitatively consistent results (Supplemental Materials S9).

The angle-specific histogram plots the number of axons directed in the corresponding angle (within the range of 2°), divided by the total number of axons detected in the image. For the total range of 180°, there are therefore 90 lines, resulting in an average amount of 1.1%. A peak was observed in the highest number of features with directionality around 88°–94° for NG-90 MEA and 37°–45° for NG-45 MEA. In contrast, the histogram of the highest number of features with directionality in culture on flat NOA81 MEA (or bare MEAs) showed no specific peak. These orientation histograms represent qualitative observations based on one representative image per condition and are not intended as quantitative comparisons across samples. The nanogroove dimensions used here were selected because they have previously been shown to induce robust neurite alignment in SH-SY5Y and hiPSC-derived neuronal cultures, providing a reliable reference topography for evaluating the feasibility of integrating nanotopography with an MEA-based functional readout.

To obtain a better understanding of the alignment of neurite outgrowths, the alignment of the features was calculated as the accumulation of the percentage of all neurites within the range of ±30° in the corresponding angle, and is presented in Figure 5d–f. In other words, alignment denotes the cumulative total amount of 15 bars before and after the corresponding angle, resulting in an average alignment of 33.3%. In the culture on NG-90 MEA, the alignment of the neuronal extension at the specific angle of 85.4°, which corresponds to the direction of nanogrooves on the MEA plate as measured in Supplemental Materials S7, is found to be 40.9%, as presented in Figure 5d. Similarly, in the NG-45 MEA culture, the alignment of the axonal extension at 44.5° is determined to be 43.6%, as can be seen in Figure 5e. These results indicate that while there is some alignment of neurites with the nanogrooves, the alignment is not particularly strong, suggesting that other factors may also play a role in directing neurite growth. A stronger alignment might be achieved with nanogrooves that have greater depth or more pronounced features, which could more significantly influence neurite organization. Further research is needed to explore how variations in nanogroove patterns could enhance alignment and better understand the mechanisms underlying neuronal network activity.

The immunostaining images of Ngn2+ iNeuron cultures on both nanogroove-modified MEAs confirmed that alignment can be observed. The alignment of the neurite outgrowths with the direction of the nanogrooves exceeds the average alignment of 33.3%. In contrast, the alignment percentage in the culture on flat NOA81 on MEA, as shown in Figure 5f, is observed to be distributed nearly uniformly within the range of 30.7% to 35.4%.

The ability of the nanogrooved platform to guide axonal alignment also suggests its potential utility for more complex in vitro models, including neuron–glia co-culture systems. In the nervous system, interactions between neurons and glial cells are essential for network development and function. The controlled topographical cues provided by the nanogrooves may facilitate the spatial organization of both neuronal and glial components, thereby broadening the applicability of this platform for studying more physiologically relevant neural models.

### 3.5. Spike and Burst Analyses

**Figure 6:**
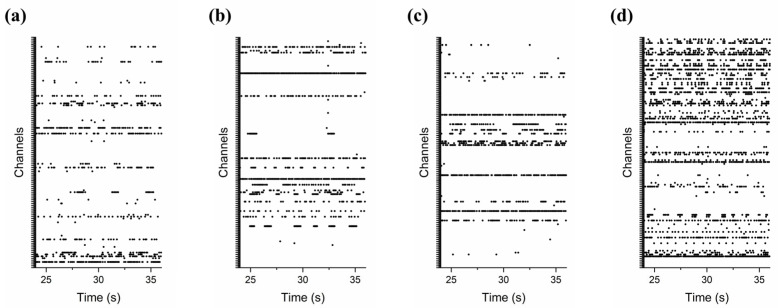
Raster plots of neuronal activity across MEA types. Electrophysiology of Ngn2+ iNeuron cultures at 18 DIV, representative examples of raster plots for all 120 channels, displaying an interval of 10 s from the middle of the third one-minute recording for the (**a**) bare MEA, (**b**) flat NOA81 MEA, (**c**) NG-90 MEA, and (**d**) NG-45 MEA.

To examine the impact of nanogrooves on the activity of Ngn2+ iNeurons, electrophysiological signals from cultures on surface-modified and bare MEA plates were recorded from the same cell culture batch using the MEA-2100 system. The resulting Day 18 in vitro data were subsequently analyzed with the MC Analyzer and Python as described in the Methods Section 2.6 and Section 2.7. All four culture configurations displayed a vivid electrophysiology as presented by example in Figure 6a–d. The presence of sustained spiking and bursting activity indicates functional development of the neuronal networks and serves as an indirect confirmation of cell viability over the course of the culture period. These figures display raster plots of spikes detected across all 120 channels during the middle 10 s interval of the third out of five consecutive one-minute recordings. Supplementary Materials S10 depicts the raster plots for the full set of 120 electrodes per configuration. Qualitative inspection of the raster plots suggests that specifically NG-45 MEA cultures (Figure 4b) exhibited higher apparent neuronal activity (Figure 6d and Figure S9d in Supplementary Materials) compared to the other culture configurations (Figure 6a–d); however, these observations are descriptive in nature and based on one sample per condition.

**Figure 7:**
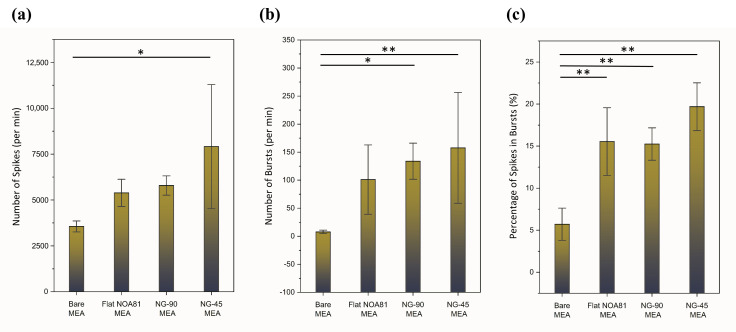
Quantitative electrophysiological metrics of neuronal cultures. Analysis of electrophysiology of Ngn2 iNeuron cultures at 18 DIV, averaged across five consecutive one-minute recordings for one sample of each MEA: (**a**) number of spikes, (**b**) number of bursts, and (**c**) percentage of spikes in bursts, in one minute as an average. The data are presented as avg ± SD. Statistical significance is shown by * for *p* < 0.05 and ** for *p* < 0.01.

To conduct a detailed quantitative analysis of the neural network activity in the four distinct cell cultures, several parameters were investigated, including the number of spikes, bursts and network bursts, and the percentage of in-burst spikes. Measurements were acquired in one-minute intervals over five consecutive time points for each of the culture configurations (one sample of each MEA configuration), with a 5 min delay for the first recording after taking out the plate from the incubator and mounting it into the head stage of the reader system. The five recordings were taken right after each other with only a few seconds in between the recordings. Performing five one-minute recordings instead of one continuous five-minute recording assisted us in data handling and archiving the data, since these files are extremely large and not easy to manipulate for further analysis. The resulting mean values and standard deviations are displayed in Figure 7. In all four cultures, the recorded signals displayed the presence of three types of negative spikes—monophasic, biphasic, and triphasic—indicative of connectivity in the neural network, in agreement with the findings by Wheeler and Nam [52].

Notably, despite the large error bars, a significantly increased spike number was observed for NG-45 compared to flat-NOA81 and NG-90 MEA plates, highlighting that nanogrooves on MEA plates can affect the neural activity of cultured Ngn2+ iNeurons (Figure 7a). A similar phenomenon is also evident in the number of bursts per minute, as illustrated in Figure 7b. Remarkably, the value is statistically significant compared to the number of bursts detected in the bare MEA culture.

In addition, the presence of either flat NOA81 or nanogroove NOA81 also significantly increased the in-burst spike ratio compared to bare MEA (Figure 7c). This finding suggests that NOA81 as a culture material is favored over the bare MEA surface by Ngn2+ iNeurons.

In relation to the occurrence of network bursts in the four distinct cultures, employing the settings outlined in Section 2.6 for detecting such bursts, no observable network bursts were identified within the cultures across the four MEAs, except for a singular instance in the third measurement of the NG-90 MEA. The lack of network bursts can probably be linked to the developmental immaturity of the neuronal cultures. Maintaining iNeurons for a longer time in culture or introducing astrocytes to the culture could potentially enhance maturation [53]. Consequently, beyond this exploratory study showcasing the utility of nanogroove-modified MEAs as NoC models, a thorough characterization involving more experimental replicas conducted on fully matured neuronal cultures is imperative to confirm the finding that nanogroove-stratified neural networks exhibit higher neural activity. Such future research could facilitate the derivation of conclusive insights regarding the functional differences across cell types and their behavioral patterns within networks derived in nanogroove-enriched culture conditions.

## 4. Conclusions

In summary, the incorporation of physical nanoscale guidance cues with cell culture plates can influence human pluripotent stem cell-derived neural network topology. The nanogrooves investigated in this study instructed the neurite outgrowth of Ngn2+ iNeurons to align with the direction of the NOA81 nanogrooves and resulted in an increased spike rate for these culture conditions. The alignment of Ngn2 cells’ axonal processes was observed to be preferentially oriented in the direction of the nanogrooves, indicating a specific guidance effect of the nanogrooves on the development and organization of the neuronal network. To enable such measurements, we successfully utilized a combined process of photolithography and microtransfer molding to generate a NOA81 nanogroove pattern onto MEA plates without jeopardizing their electrical functionality, as confirmed by light microscopy, scanning electron microscopy, and atomic force microscopy, as well as the actual MEA recordings.

## Data Availability

All data supporting the findings of this study are available within the paper and Supplementary Materials or upon reasonable request from the corresponding author.

## Supplementary Materials

The following supporting information can be downloaded at: https://www.mdpi.com/article/10.3390/mi17050524/s1. Photomask design for NOA81 photolithography on MEA, nanogroove orientations on MEA, details of the mask aligning and washing/drying steps, incorporation of a PMMA ring, Multi Channel Analyzer experimental setup, filtered data in Multi Channel Analyzer, fabrication of nanogrooves onto MEA plate using combined microtransfer molding and photolithography, control culture of Ngn2+ iNeurons on conventional substrates, structure-tensor-based validation of neurite alignment, and raster plots of spike events. Figure S1: The layout of (a–c) different magnifications of a standard MEA (120MEA200/30iR-Ti-w/o) and (d,e) the designed photomask to cover 120 recording electrodes and 4 reference electrodes of the MEA during the exposure step. Figure S2: Aligning the grooves orientation with respect to the direction of the electrodes, in (a), the grooves are in the direction of the electrodes (90° orientation), and in (b), the grooves are in diagonal orientation with respect to the direction of the electrodes (45° orientation). The lines represent the grooves, but they are not drawn to scale. Figure S3: Schematic of the incorporating procedure: (i) A PMMA ring with a height of 6 mm and inner and outer diameters of 19 mm and 24 mm, respectively, was prepared. (ii) A small amount of PDMS was uniformly applied with a height of approximately 2 mm on the top surface of the ring. (iii) The ring was flipped over and placed in the center of the MEA substrate, and additional PDMS was applied around the ring’s perimeter. Figure S4: Experimental setup configuration used in the Multi Channel Analyzer software. Figure S5: (a) Filtered data recorded at Channel D11 from Ngn2 iNeurons cell culture on the NG-45 MEA in the first 10 s of a one-minute recording. (b) The cutouts of the detected spikes in that time interval. Figure S6: (a) MEA assembled with PMMA ring, and (b) the transferred nanogroove patterns (also pointed by the arrow), visible by appearing colors due to light interference on the surface of a standard MEA. (c) The NOA81 layer on the MEA surface, leaving the recording electrodes uncovered. (d) Optical image of a single electrode of an MEA with NOA81 nanogrooves. The grooves are clearly visible (also pointed by the arrows) for an alignment of (e) 90° orientation (NG-90 MEA) and (f) 45° orientation (NG-45 MEA) (scale bars 20 μm). Figure S7: Representative images of Ngn2+ iNeuron cultures grown on conventional 24-well plates. (a) SMI-312 staining (axonal neurofilament marker), (b) MAP2 staining (microtubule-associated protein 2), (c) merged image (DAPI, MAP2, and SMI-312), and (d) phase-contrast image. DAPI (blue) indicates cell nuclei, MAP2 (red) highlights neuronal cell bodies and dendrites, and SMI-312 (green) labels axonal projections. Figure S8: Directional neurite growth analysis on MEAs using structure-tensor-based orientation analysis (OrientationJ). Distribution of neurite orientations across the electrode regions of Ngn2+ iNeuron cultures on (a) NG-90 MEA, (b) NG-45 MEA, and (c) flat NOA81 MEA. Figure S9: Raster plots of spikes detected in 1 min measurement of Ngn2 iNeuron cultures at 18 DIV on (a) bare MEA, (b) flat NOA81 MEA, (c) NG-90 MEA, and (d) NG-90 MEA.

## Abbreviations

The following abbreviations are used in this manuscript:

NoC	Nervous System-on-Chip
BoC	Brain-on-Chip
μTM	Microtransfer Molding
ECM	Extracellular Matrix
MEA	Microelectrode Array
hiPSC	Human Induced Pluripotent Stem Cell
IPA	Isopropanol
PLO	Poly-L-Ornithine
DIV	Day In Vitro
NOA81	Norland Optical Adhesive 81
NG	Nanogroove
PDMS	Polydimethylsiloxane
COC	Cyclic Olefin Copolymer
SEM	Scanning Electron Microscopy
AFM	Atomic Force Microscopy

## References

[1] Frimat J.-P., Luttge R. (2019). The Need for Physiological Micro-Nanofluidic Systems of the Brain. Front. Bioeng. Biotechnol..

[2] Amirifar L., Shamloo A., Nasiri R., de Barros N.R., Wang Z.Z., Unluturk B.D., Libanori A., Ievglevskyi O., Diltemiz S.E., Sances S. (2022). Brain-on-a-Chip: Recent Advances in Design and Techniques for Microfluidic Models of the Brain in Health and Disease. Biomaterials.

[3] Nikolakopoulou P., Rauti R., Voulgaris D., Shlomy I., Maoz B.M., Herland A. (2020). Recent Progress in Translational Engineered *in Vitro* Models of the Central Nervous System. Brain.

[4] Nandi S., Ghosh S., Garg S., Sarkar A., Ghosh S. (2022). Brain-on-a-Chip. Microfluidics and Multi Organs on Chip.

[5] Walczak P.A., Perez-Esteban P., Bassett D.C., Hill E.J. (2021). Modelling the Central Nervous System: Tissue Engineering of the Cellular Microenvironment. Emerg. Top. Life Sci..

[6] Holloway P.M., Willaime-Morawek S., Siow R., Barber M., Owens R.M., Sharma A.D., Rowan W., Hill E., Zagnoni M. (2021). Advances in Microfluidic *in Vitro* Systems for Neurological Disease Modeling. J. Neurosci. Res..

[7] Tan H.-Y., Cho H., Lee L.P. (2020). Human Mini-Brain Models. Nat. Biomed. Eng..

[8] Jorfi M., D’Avanzo C., Kim D.Y., Irimia D. (2018). Three-Dimensional Models of the Human Brain Development and Diseases. Adv. Healthc. Mater..

[9] Luttge R. (2022). Nanofabricating Neural Networks: Strategies, Advances, and Challenges. J. Vac. Sci. Technol. B.

[10] Omidi S., Berdichevsky Y. (2025). Pathway-like Activation of 3D Neuronal Constructs with an Optical Interface. Biosensors.

[11] Jain D., Mattiassi S., Goh E., Yim E.K. (2020). Extracellular Matrix and Biomimetic Engineering Microenvironment for Neuronal Differentiation. Neural Regen. Res..

[12] Wilems T., Vardhan S., Wu S., Sakiyama-Elbert S. (2019). The Influence of Microenvironment and Extracellular Matrix Molecules in Driving Neural Stem Cell Fate within Biomaterials. Brain Res. Bull..

[13] Yang C.-Y., Huang W.-Y., Chen L.-H., Liang N.-W., Wang H.-C., Lu J., Wang X., Wang T.-W. (2021). Neural Tissue Engineering: The Influence of Scaffold Surface Topography and Extracellular Matrix Microenvironment. J. Mater. Chem. B.

[14] Bucaro M.A., Vasquez Y., Hatton B.D., Aizenberg J. (2012). Fine-Tuning the Degree of Stem Cell Polarization and Alignment on Ordered Arrays of High-Aspect-Ratio Nanopillars. ACS Nano.

[15] Onesto V., Cancedda L., Coluccio M.L., Nanni M., Pesce M., Malara N., Cesarelli M., Di Fabrizio E., Amato F., Gentile F. (2017). Nano-Topography Enhances Communication in Neural Cells Networks. Sci. Rep..

[16] Eftekhari B.S., Eskandari M., Janmey P.A., Samadikuchaksaraei A., Gholipourmalekabadi M. (2020). Surface Topography and Electrical Signaling: Single and Synergistic Effects on Neural Differentiation of Stem Cells. Adv. Funct. Mater..

[17] Bugnicourt G., Brocard J., Nicolas A., Villard C. (2014). Nanoscale Surface Topography Reshapes Neuronal Growth in Culture. Langmuir.

[18] Xie S., Luttge R. (2014). Imprint Lithography Provides Topographical Nanocues to Guide Cell Growth in Primary Cortical Cell Culture. Microelectron. Eng..

[19] Teo B.K.K., Wong S.T., Lim C.K., Kung T.Y.S., Yap C.H., Ramagopal Y., Romer L.H., Yim E.K.F. (2013). Nanotopography Modulates Mechanotransduction of Stem Cells and Induces Differentiation through Focal Adhesion Kinase. ACS Nano.

[20] Tonazzini I., Masciullo C., Savi E., Sonato A., Romanato F., Cecchini M. (2020). Neuronal Contact Guidance and YAP Signaling on Ultra-Small Nanogratings. Sci. Rep..

[21] Litowczenko J., Maciejewska B.M., Wychowaniec J.K., Kościński M., Jurga S., Warowicka A. (2019). Groove-patterned Surfaces Induce Morphological Changes in Cells of Neuronal Origin. J. Biomed. Mater. Res. A.

[22] Ristola M., Fedele C., Hagman S., Sukki L., Kapucu F.E., Mzezewa R., Hyvärinen T., Kallio P., Priimagi A., Narkilahti S. (2021). Directional Growth of Human Neuronal Axons in a Microfluidic Device with Nanotopography on Azobenzene-Based Material. Adv. Mater. Interfaces.

[23] Bastiaens A.J., Frimat J.-P., van Nunen T., Luttge R. (2019). Exploiting Nanogroove-Induced Cell Culture Anisotropy to Advance *in Vitro* Brain Models. J. Vac. Sci. Technol. B.

[24] Simitzi C., Karali K., Ranella A., Stratakis E. (2018). Controlling the Outgrowth and Functions of Neural Stem Cells: The Effect of Surface Topography. ChemPhysChem.

[25] Yoo J., Noh M., Kim H., Jeon N.L., Kim B.S., Kim J. (2015). Nanogrooved Substrate Promotes Direct Lineage Reprogramming Offibroblasts to Functional Induced Dopaminergic Neurons. Biomaterials.

[26] Cecchini M., Bumma G., Serresi M., Beltram F. (2007). PC12 Differentiation on Biopolymer Nanostructures. Nanotechnology.

[27] Ferrari A., Cecchini M., Degl’Innocenti R., Beltram F. (2009). Directional PC12 Cell Migration Along Plastic Nanotracks. IEEE Trans. Biomed. Eng..

[28] Xie S., Schurink B., Wolbers F., Luttge R., Hassink G. (2014). Nanoscaffold’s Stiffness Affects Primary Cortical Cell Network Formation. J. Vac. Sci. Technol..

[29] Bastiaens A.J., Xie S., Luttge R. (2018). Investigating the Interplay of Lateral and Height Dimensions Influencing Neuronal Processes on Nanogrooves. J. Vac. Sci. Technol. B.

[30] Xie S. (2016). Brain-on-a-Chip Integrated Neuronal Networks.

[31] Bastiaens A.J., Xie S., Luttge R. (2019). Nanogroove-Enhanced Hydrogel Scaffolds for 3D Neuronal Cell Culture: An Easy Access Brain-on-Chip Model. Micromachines.

[32] Juhász G., Mittli D., Tukacs V., Kékesi K.A. (2022). Electrophysiology and Single Cells.

[33] Sabahi-Kaviani R., Luttge R. (2021). Investigating the Pattern Transfer Fidelity of Norland Optical Adhesive 81 for Nanogrooves by Microtransfer Molding. J. Vac. Sci. Technol. B.

[34] Frega M., van Gestel S.H.C., Linda K., van der Raadt J., Keller J., Van Rhijn J.-R., Schubert D., Albers C.A., Nadif Kasri N. (2017). Rapid Neuronal Differentiation of Induced Pluripotent Stem Cells for Measuring Network Activity on Micro-Electrode Arrays. J. Vis. Exp..

[35] Shiryaeva M. (2022). Ngn2 INeurons as a Reliable Workhorse for Brain-on-Chip Development and Validation. Master’s Thesis.

[36] Wägli P., Homsy A., de Rooij N.F. (2010). Norland Optical Adhesive (NOA81) Microchannels with Adjustable Surface Properties and High Chemical Resistance against IR-Transparent Organic Solvents. Procedia Eng..

[37] Wang S., van Rhijn J.-R., Akkouh I., Kogo N., Maas N., Bleeck A., Ortiz I.S., Lewerissa E., Wu K.M., Schoenmaker C. (2022). Loss-of-Function Variants in the Schizophrenia Risk Gene SETD1A Alter Neuronal Network Activity in Human Neurons through the CAMP/PKA Pathway. Cell Rep..

[38] Frega M., Linda K., Keller J.M., Gümüş-Akay G., Mossink B., van Rhijn J.-R., Negwer M., Klein Gunnewiek T., Foreman K., Kompier N. (2019). Neuronal Network Dysfunction in a Model for Kleefstra Syndrome Mediated by Enhanced NMDAR Signaling. Nat. Commun..

[39] Sim J.H., Moon H.J., Roh Y.H., Jung H.W., Bong K.W. (2017). Fabrication of NOA Microfluidic Devices Based on Sequential Replica Molding. Korean J. Chem. Eng..

[40] Wägli P., Homsy A., de Rooij N.F. (2011). Norland Optical Adhesive (NOA81) Microchannels with Adjustable Wetting Behavior and High Chemical Resistance against a Range of Mid-Infrared-Transparent Organic Solvents. Sens. Actuators B Chem..

[41] Moonen E., Luttge R., Frimat J.-P. (2018). Single Cell Trapping by Capillary Pumping Using NOA81 Replica Moulded Stencils. Microelectron. Eng..

[42] Sabahi-Kaviani R., Luttge R. (2020). Gaining Micropattern Fidelity in an NOA81 Microsieve Laser Ablation Process. Micromachines.

[43] Sabahi-Kaviani R., van Boekel D., Luttge R. (2023). Computational Study of Mechanical Stresses in a Cell Interacting with Micromechanical Cues and Microfabrication of Such Cues in Nervous System-on-Chips. J. Vac. Sci. Technol. B.

[44] Nečas D., Klapetek P. (2012). Gwyddion: An Open-Source Software for SPM Data Analysis. Open Phys..

[45] Schindelin J., Arganda-Carreras I., Frise E., Kaynig V., Longair M., Pietzsch T., Preibisch S., Rueden C., Saalfeld S., Schmid B. (2012). Fiji: An Open-Source Platform for Biological-Image Analysis. Nat. Methods.

[46] Habets A.M.M.C., Van Dongen A.M.J., Van Huizen F., Corner M.A. (1987). Spontaneous Neuronal Firing Patterns in Fetal Rat Cortical Networks during Development in Vitro: A Quantitative Analysis. Exp. Brain Res..

[47] Regalia G., Biffi E., Ferrigno G., Pedrocchi A. (2015). A Low-Noise, Modular, and Versatile Analog Front-End Intended for Processing *In Vitro* Neuronal Signals Detected by Microelectrode Arrays. Comput. Intell. Neurosci..

[48] Watkins P.T., Santhanam G., Shenoy K.V., Harrison R.R. (2004). Validation of Adaptive Threshold Spike Detector for Neural Recording. Proceedings of the the 26th Annual International Conference of the IEEE Engineering in Medicine and Biology Society, San Francisco, CA, USA, 1–5 September 2004.

[49] Pasquale V., Martinoia S., Chiappalone M. (2010). A Self-Adapting Approach for the Detection of Bursts and Network Bursts in Neuronal Cultures. J. Comput. Neurosci..

[50] Pagan-Diaz G.J., Drnevich J., Ramos-Cruz K.P., Sam R., Sengupta P., Bashir R. (2020). Modulating Electrophysiology of Motor Neural Networks via Optogenetic Stimulation during Neurogenesis and Synaptogenesis. Sci. Rep..

[51] Bastiaens A.J., Xie S., Mustafa D.A.M., Frimat J.-P., den Toonder J.M.J., Luttge R. (2018). Validation and Optimization of an Image-Based Screening Method Applied to the Study of Neuronal Processes on Nanogrooves. Front. Cell. Neurosci..

[52] Wheeler B.C., Nam Y. (2011). In Vitro Microelectrode Array Technology and Neural Recordings. Crit. Rev. Biomed. Eng..

[53] Ho S.M., Hartley B.J., Tcw J., Beaumont M., Stafford K., Slesinger P.A., Brennand K.J. (2016). Rapid Ngn2-Induction of Excitatory Neurons from HiPSC-Derived Neural Progenitor Cells. Methods.

