# Supplementary material for: Nanogroove-Induced Enhancement of Neural Spike Activity in Stem Cell-Derived Networks
Source: Micromachines (Basel). 2026 Apr 25;17(5):524. doi: 10.3390/mi17050524 (PMC13209113; doi:10.3390/mi17050524)
Supplement: Supplementary file 1 [file micromachines-17-00524-s001.zip › micromachines-4247580-supplementary.pdf]

Nanogroove-Induced Enhancement of Neural Spike Activity in Stem Cell-Derived Networks

Rahman Sabahi-Kaviani [1], Marina Shiryaeva [1,†], and Regina Luttge [1,2,*]

[1] Neuro-Nanoscale Engineering, Department of Mechanical Engineering/Microsystems and Institute of Complex Molecular Systems, Eindhoven University of Technology, 5600 MB Eindhoven, The Netherlands; r.sabahi.kaviani@tue.nl
[2] Eindhoven Artificial Intelligence Systems Institute and Casimir Institute, Eindhoven University of Technology, 5600 MB Eindhoven, The Netherlands.

[†] Current address: Center for Neurogenomics and Cognitive Research (CNCR), Vrije Universiteit (VU) Amster-dam, 1081 HV Amsterdam, The Netherlands.
[*] Correspondence: r.luttge@tue.nl

S1. Photomask design for NOA81 photolithography on MEA

The layout of a standard MEA (120MEA200/30iR-Ti-w/o, Multi Channel Systems MCS GmbH, Reutlingen, Germany) is shown in Fig. S1. It consists of 120 recording electrodes and four reference electrodes, with a recording electrode spacing of 200 µm and an electrode diameter of 30 µm (Fig. S1a-c). To mask the UV light during the NOA81 curing step, a custom foil photomask was designed (in AutoCAD 2022, printed by CAD/Art Services Inc., Bandon, OR, United States) and utilized with the same layout as the electrodes on the MEA but with circles of a slightly larger diameter than the electrode, i.e. 40 µm (Fig. S1d-e).

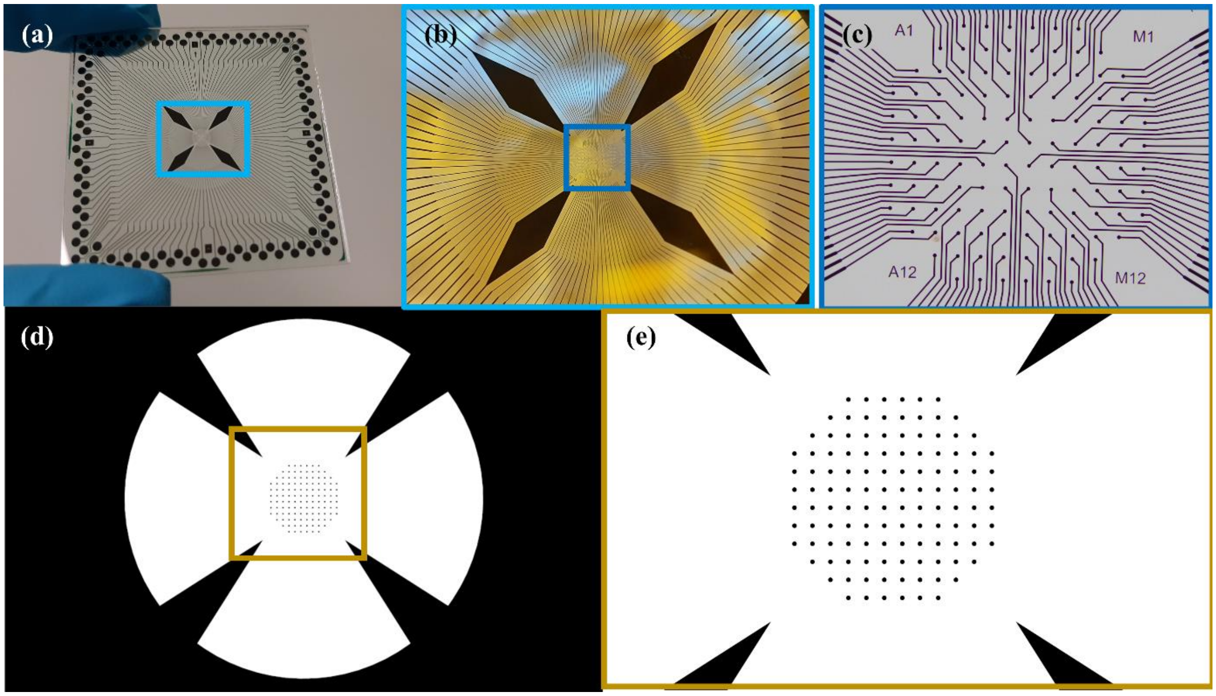

FIG. S1. The layout of (a-c) different magnifications of a standard MEA (120MEA200/30iR-Ti-w/o) and (d-e) the designed photomask to cover 120 recording electrodes and 4 reference electrodes of the MEA during exposure step.

S2. Nanogroove orientations on MEA

Two distinguished orientations of the nanogrooves relative to the direction of the electrodes, were selected, i.e. 90˚ alignment, NG-90 MEA (nanogrooves running parallel to the electrodes) and 45˚ alignment, NG-45 MEA (nanogrooves running diagonally relative to the electrodes) (Fig. S2). Additionally, a flat PDMS was used to create a flat NOA81 layer on the MEA, as a control experiment.

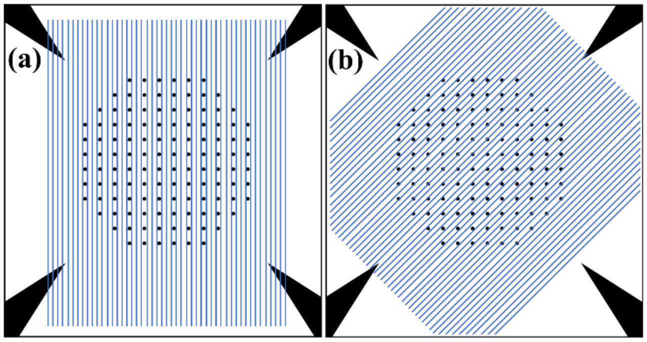

FIG. S2. Aligning the grooves orientation with respect to direction of the electrodes, in (a) the grooves are in the direction of electrodes (90˚ orientation) and in (b) the groves are in diagonal ordination with respect to the direction of the electrodes (45˚ orientation). The lines represent the grooves, but they are not drawn to scale.

S3. Details of the mask aligning and washing/drying steps

The MEA, activated by plasma asher, and the photomask-PDMS mold assembly, which had been spin-coated with NOA81, were positioned in a mask aligner (MJB4 Mask Aligner, SÜSS MicroTec SE, Germany). The table with the MEA atop was gradually raised reaching as small distance to the photomask as possible but just not yet contacting the wet NOA81 film.

Subsequently, the 120 electrodes on the MEA were aligned with the corresponding pattern on the photomask. When contact between MEA and NOA81 film was established, it was important to ensure that the NOA81 was completely coated on the surface of the MEA, which was confirmed by changing the field of view in the microscope of the mask aligner.

Then, the NOA81 layer was subjected to ultraviolet (UV) exposure energy of 670 mJ/cm^2 through the photomask to initiate curing. After exposure, the Photomask-PDMS mold assembly was carefully peeled from the MEA from one corner using a tweezer. The MEA was then placed in an acetone dish and gently shaken for 5 seconds to wash out the uncured NOA81, followed by a 10 s gentle shake in an isopropanol (IPA, Article number

76051455.9010, Boom B.V., the Netherlands) dish. The nanogroove-modified MEA was dried with an air gun in a direction normal to the MEA.

The patterns were inspected for uncured NOA81 residue by simple light microscopy (Olympus SZ61 Stereo Microscope was used for this judgment). If additional washing steps were required, the process of acetone and IPA washing, and drying, was repeated. When the features appeared clearly without residue, the MEA was subjected to an additional UV light exposure via the UV-LED exposure system (IDONUS, UV-EXP 150R, Neuchatel, Switzerland) with an energy dosage of 8000 mJ/cm^2 at the intensity of 15 mW/cm^2 to fully cure the NOA81 film (nanogroove or flat).

S4. Incorporation of a PMMA ring

Based on the dimensions of the glass rings (standard type) generally known to be mounted on a 120MEA200/30iR-Ti-gr (standard MEA ring details in Multichannel Systems website[1]), we made a ring from polymethylmethacrylate (PMMA) from a 6 mm thick sheet material (ERIKS, the Netherlands) and affixed it to our surface modified MEA to create an environment for the cultivation and growth of cells. The PMMA ring was fabricated using a laser cutting system (Universal Laser System, VLS Model 3.50, ENGRAVING SYSTEMS, LLC, Connecticut, USA). Obeying the dimensions of Multi Channel Systems, we made a CAD drawing of the ring with an inner diameter of 19 mm, and an outer diameter of 24 mm (standard MEA ring details in Multichannel Systems website) to be used to control the cutter. PDMS, as prepared in Sec. 2.3.1 in the main text of the paper, was used as an adhesive to bond the PMMA ring to the MEA substrate. Firstly, a controlled and minimal amount of PDMS (approximately 1–2 mm in height) was uniformly applied to the top surface of the ring. Then the ring was placed in the center of the MEA substrate, and if needed a small additional PDMS was applied around the ring's perimeter to reinforce bonding. The PDMS was subsequently cured in an oven at 65 ºC for 2 h. A schematic of the procedure is depicted in Fig. S3.

---

[1] https://www.multichannelsystems.com/sites/multichannelsystems.com/files/documents/data_sheets/MCS_MEA%20Ring%20Options.pdf

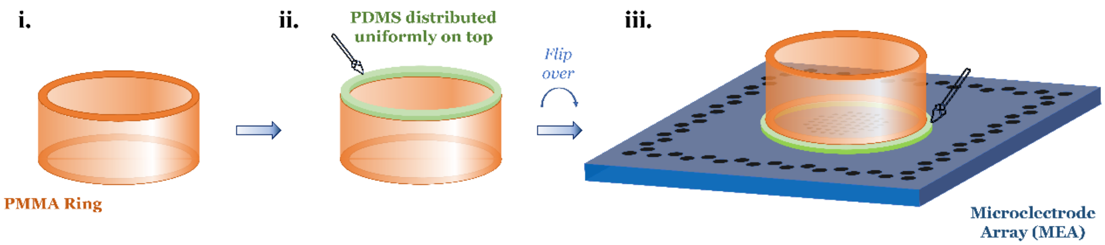

FIG. S3. Schematic of the incorporating procedure: (i) PMMA ring with a height of 6 mm and inner and outer diameters of 19 mm and 24 mm, respectively, was prepared. (ii) A small amount of PDMS was uniformly applied with a height of approximately 1-2 mm on the top surface of the ring. (iii) The ring was flipped over and placed in the center of the MEA substrate and if needed, a small additional PDMS was applied around the ring's perimeter.

S5. Multi Channel Analyzer experimental setup

The experimental configurations employed in Multi Channel Analyzer software (Multi Channel Systems MCS GmbH, Reutlingen, Germany) is outlined in Fig. S4.

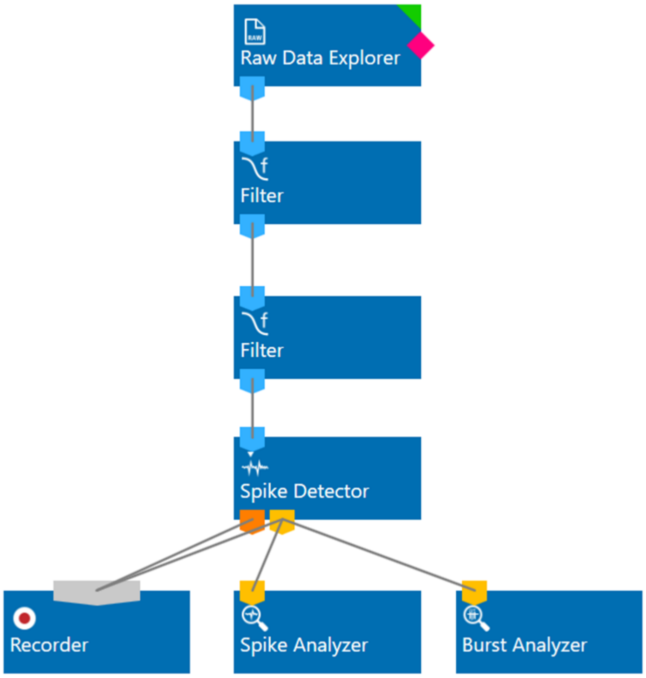

FIG. S4. Experimental setup configuration used in Multi Channel Analyzer software.

S6. Filtered data in Multi Channel Analyzer

An example of filtered data recorded from Ngn2 iNeurons cell culture on the NG-45 MEA (Channel D11) is presented in Fig. S5a. The detected spikes are depicted in Fig. S5b through cutouts. Baseline noise for each channel was automatically estimated by the Multi Channel Analyzer software from a short predefined recording window, and the spike detection threshold was set to five times the noise level.

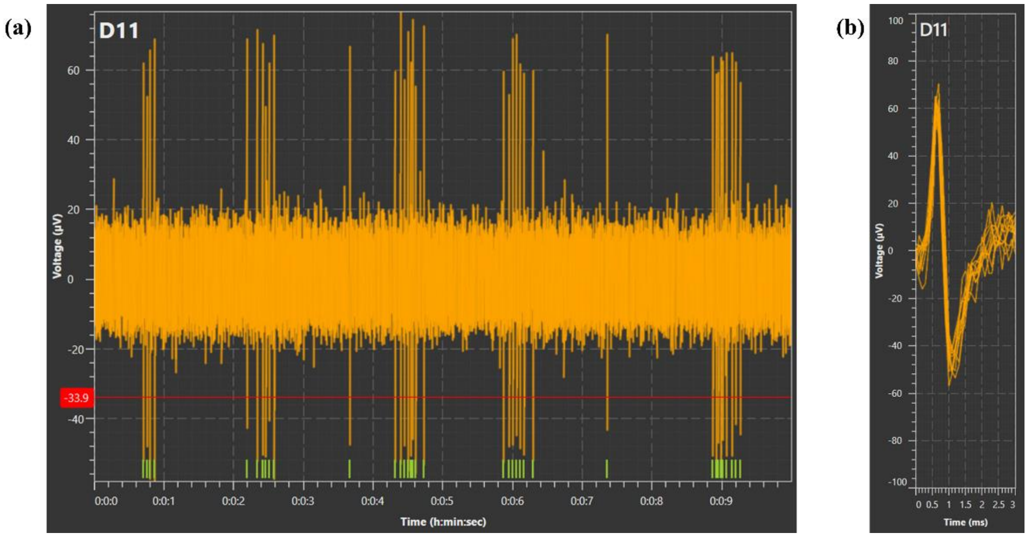

FIG. S5. (a) Filtered data recorded at Channel D11 from Ngn2 iNeurons cell culture on the NG-45 MEA in the first 10 s of a one minute recording. (b) The cutouts of the detected spikes in that time interval.

S7. Fabrication of nanogrooves onto MEA plate using combined microtransfer molding and photolithography

To obtain quantitative information on the impact of nanogrooves on neuronal differentiation, organization and activity, MEA plates with either flat or nanogroove NOA81 were prepared by microtransfer molding (Sec. 2.3.2 in the main text of the

paper). The presence of nanogrooves on the MEA surface was confirmed through visual inspection under both light interference and microscopy (Figs. S6b-d). The microscopic images of a single electrode on a MEA incorporated with NOA81 nanogrooves (Fig. S6d) confirmed the successful implementation of our fabrication technique consisting of the combined process of simultaneously performing µTM and UV photolithography. Using this process, only the area between the electrodes were covered by the nanopatterned NOA81, which was also confirmed by signal recordings in culture. For the manually assembly of photomask and PDMS molds aligned by eye, we confirmed the presence of nanogrooves in either 90˚ or 45˚ alignment to the electrode array through Figs. S6e and S6f with measured angles of 85.4˚ (NG-90) and 44.5˚ (NG-45), respectively. If a more precise alignment is critical, it can be achieved through improved placement of the PDMS mold onto the photomask using a microscope and a micromanipulator table, similar to the mask aligner systems we used for the µTM of the pattern to the MEA plate.

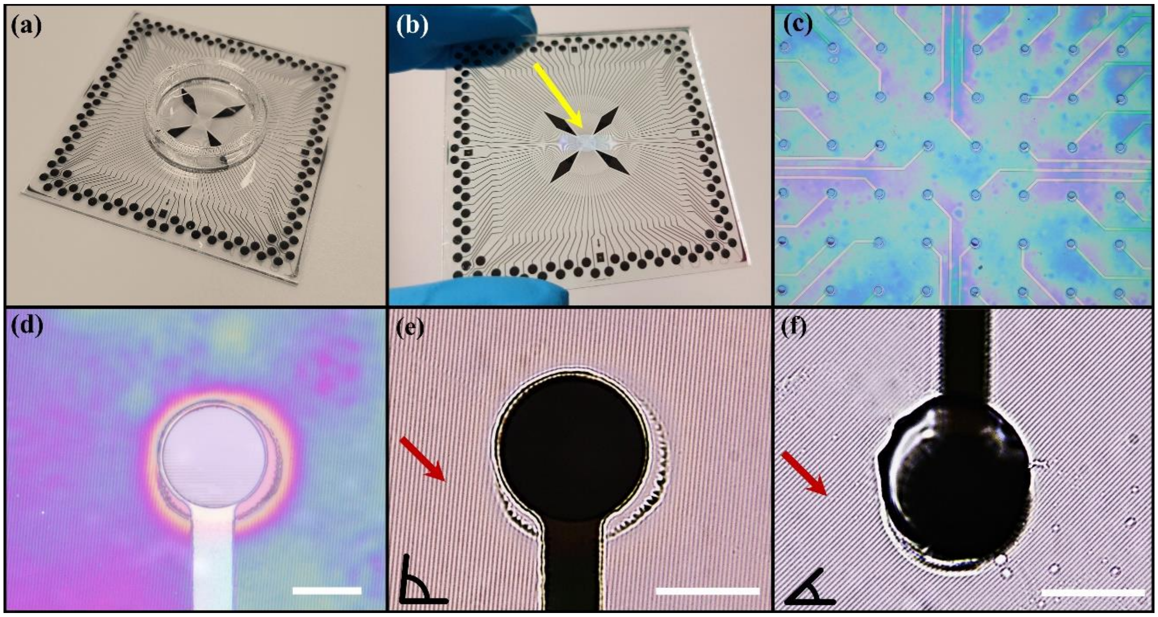

FIG. S6. (a) MEA assembled with PMMA ring, (b) the transferred nanogroove patterns are visible (also pointed by the arrow) by appearing colors due to light interference on the surface of a standard MEA. (c) NOA81 layer on the MEA surface leaving the recording electrodes uncovered. (d) Optical image of a single electrode of a MEA with NOA81 nanogrooves. The grooves are clearly visible (also pointed by the arrows) for an

alignment of (e) 90˚ orientation (NG-90 MEA) and (f) 45˚ orientation (NG-45 MEA) (scale bars 20 μm).

S8. Control culture of Ngn2+ iNeurons on conventional substrates

To provide a reference for neuronal morphology under standard culture conditions, Ngn2+ iNeurons were cultured on conventional 24-well plates and analyzed after differentiation. Immunostaining was performed to visualize neuronal structures, including dendrites (MAP2), axons (SMI-312), and cell nuclei (DAPI).

The cultures exhibited well-developed neuronal morphology, with MAP2-positive cell bodies and dendritic networks, and SMI-312-positive axonal projections extending across the culture surface. The merged fluorescence images confirm the presence of neuronal networks, while phase-contrast imaging illustrates overall cell distribution and morphology (Fig. S7). These control cultures serve as a qualitative reference for comparison with neurons grown on NOA81-modified MEA platforms.

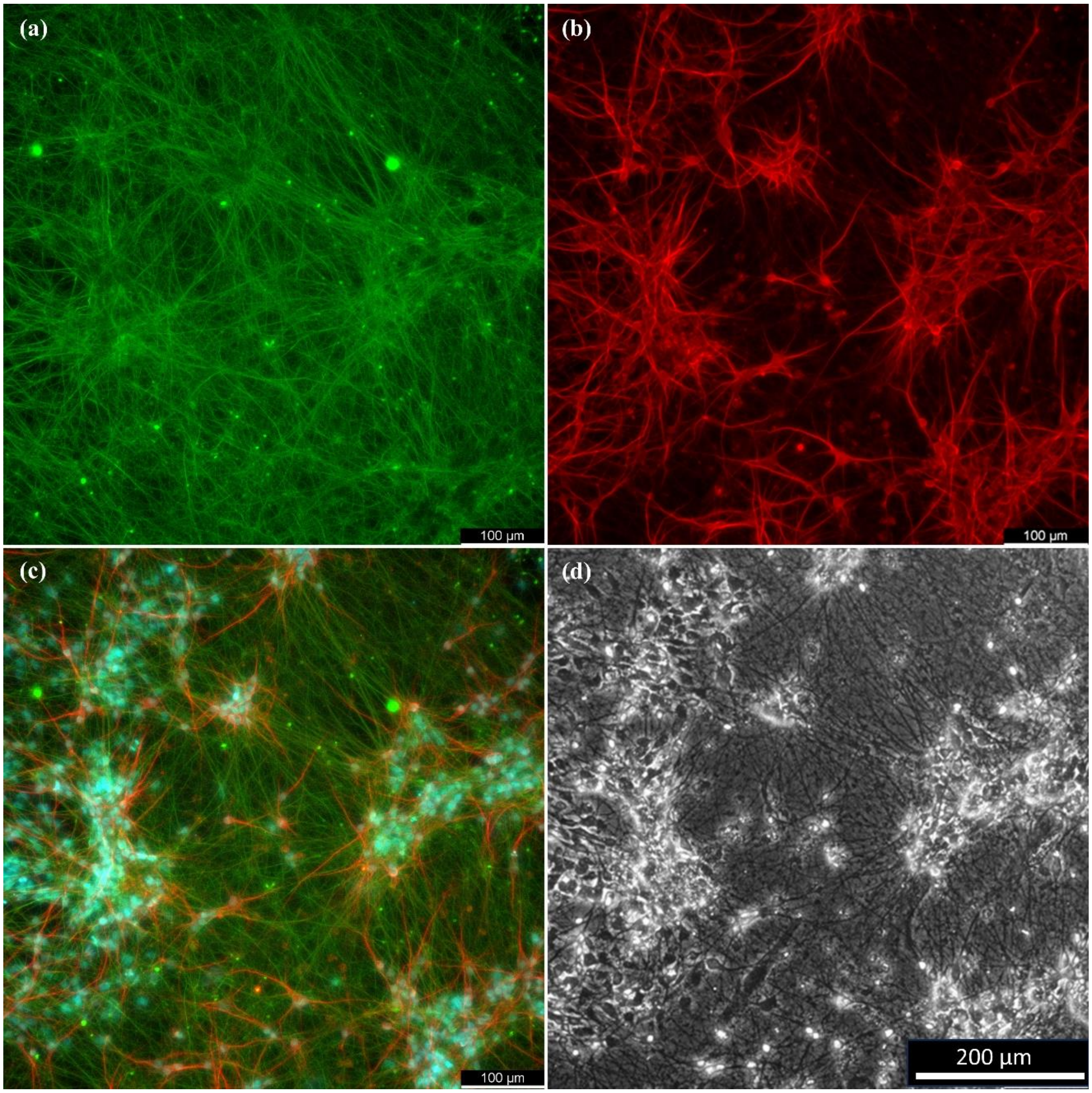

FIG. S7. Representative images of Ngn2+ iNeuron cultures grown on conventional 24-well plates. (a) SMI-312 staining (axonal neurofilament marker), (b) MAP2 staining (microtubule-associated protein 2), (c) merged image (DAPI, MAP2, and SMI-312), and (d) phase-contrast image. DAPI (blue) indicates cell nuclei, MAP2 (red) highlights neuronal cell bodies and dendrites, and SMI-312 (green) labels axonal projections.

S9. Structure-tensor–based validation of neurite alignment

To independently validate the neurite alignment observed using Fourier-based directionality analysis, an additional orientation analysis was performed using the OrientationJ plugin in FIJI. OrientationJ employs a structure-tensor–based approach that estimates local feature orientation from image intensity gradients and is less sensitive to large, highly oriented non-cellular structures such as MEA electrode traces. Orientation analysis of SMI 312–stained axons revealed preferred orientations aligned with the nanogroove direction on both NG-90 and NG-45 MEAs (Fig. S8a,b). In contrast, cultures on flat NOA81 MEAs exhibited less dominant orientation (Fig. 8c).

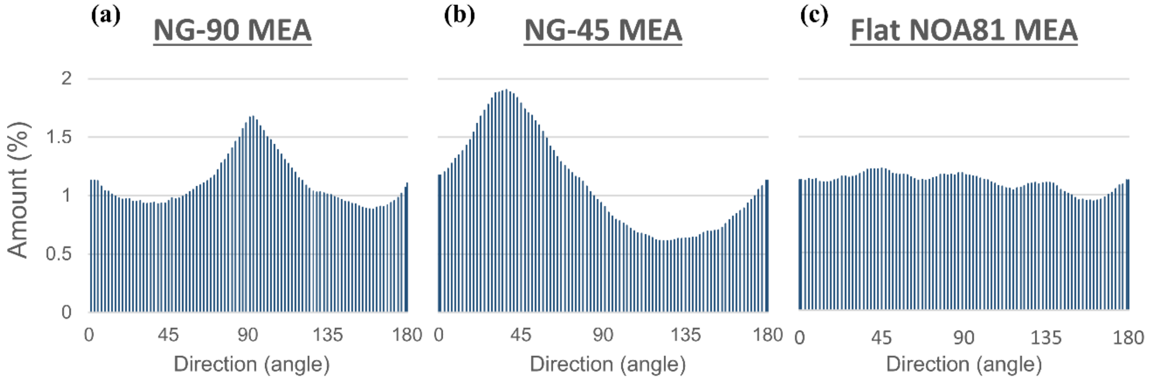

FIG. S8. Directional neurite growth analysis on MEAs using structure-tensor–based orientation analysis (OrientationJ). Distribution of neurite orientations across the electrode regions of Ngn2+ iNeuron cultures on (a) NG-90 MEA, (b) NG-45 MEA, and (c) flat NOA81 MEA.

S10. Raster plots of spikes events

The raster plots of the spikes detected in the Ngn2 iNeurons cultures on 4 different MEA plates during the 1-min measurement are shown in Fig. S9.

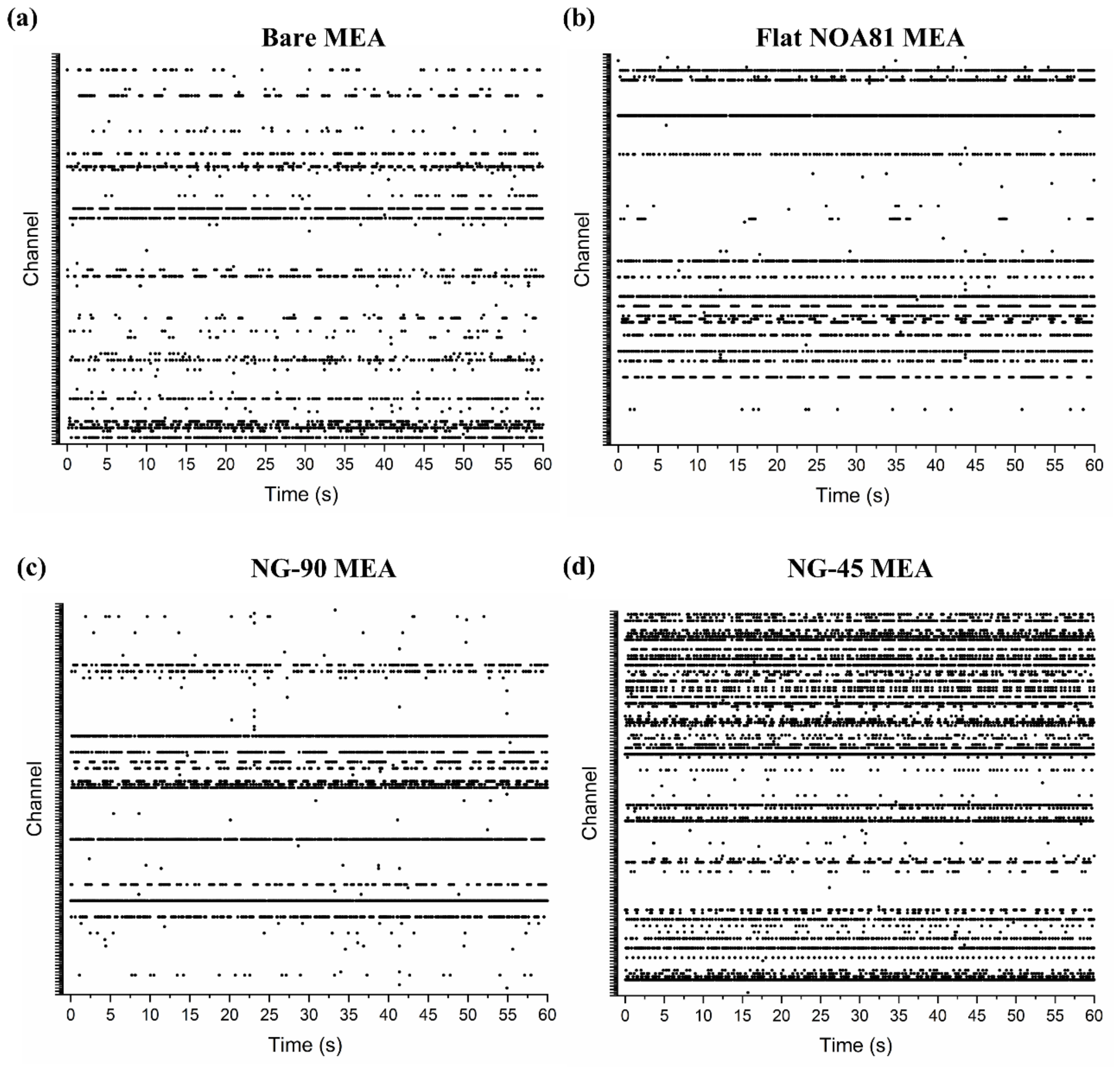

FIG. S9. Raster plots of spikes detected in 1-min measurement of Ngn2 iNeurons cultures at 18 DIV on (a) bare MEA, (b) flat NOA81 MEA, (c) NG-90 MEA, and (d) NG-90 MEA.
